# *Bacillus anthracis *in China and its relationship to worldwide lineages

**DOI:** 10.1186/1471-2180-9-71

**Published:** 2009-04-15

**Authors:** Tatum S Simonson, Richard T Okinaka, Bingxiang Wang, W Ryan Easterday, Lynn Huynh, Jana M U'Ren, Meghan Dukerich, Shaylan R Zanecki, Leo J Kenefic, Jodi Beaudry, James M Schupp, Talima Pearson, David M Wagner, Alex Hoffmaster, Jacques Ravel, Paul Keim

**Affiliations:** 1 Department of Biological Sciences, Northern Arizona University, Flagstaff, AZ 86011-5640, USA; 2 Bioscience Division, Los Alamos National Laboratory, Los Alamos New Mexico, 87545, USA; 3 Lanzhou Institute of Biological Product, Lanzhou, PR China; 4 Epidemiologic Investigations Laboratory, Center for Disease Control and Prevention, Atlanta, GA 30333, USA; 5 The J Craig Venter Institute, Rockville, Maryland, USA; 6 Pathogen Genomics Division, Translational Genomics Research Institute, Pathogen Genomics Division, 445 N Fifth Street, Phoenix, AZ 85004, USA

## Abstract

**Background:**

The global pattern of distribution of 1033 *B. anthracis *isolates has previously been defined by a set of 12 conserved canonical single nucleotide polymorphisms (canSNP). These studies reinforced the presence of three major lineages and 12 sub-lineages and sub-groups of this anthrax-causing pathogen. Isolates that form the A lineage (unlike the B and C lineages) have become widely dispersed throughout the world and form the basis for the geographical disposition of "modern" anthrax. An archival collection of 191 different *B. anthracis *isolates from China provides a glimpse into the possible role of Chinese trade and commerce in the spread of certain sub-lineages of this pathogen. Canonical single nucleotide polymorphism (canSNP) and multiple locus VNTR analysis (MLVA) typing has been used to examine this archival collection of isolates.

**Results:**

The canSNP study indicates that there are 5 different sub-lineages/sub-groups in China out of 12 previously described world-wide canSNP genotypes. Three of these canSNP genotypes were only found in the western-most province of China, Xinjiang. These genotypes were A.Br.008/009, a sub-group that is spread across most of Europe and Asia; A.Br.Aust 94, a sub-lineage that is present in Europe and India, and A.Br.Vollum, a lineage that is also present in Europe. The remaining two canSNP genotypes are spread across the whole of China and belong to sub-group A.Br.001/002 and the A.Br.Ames sub-lineage, two closely related genotypes. MLVA typing adds resolution to the isolates in each canSNP genotype and diversity indices for the A.Br.008/009 and A.Br.001/002 sub-groups suggest that these represent older and established clades in China.

**Conclusion:**

*B. anthracis *isolates were recovered from three canSNP sub-groups (A.Br.008/009, A.Br.Aust94, and A.Br.Vollum) in the western most portion of the large Chinese province of Xinjiang. The city of Kashi in this province appears to have served as a crossroads for not only trade but the movement of diseases such as anthrax along the ancient "silk road". Phylogenetic inference also suggests that the A.Br.Ames sub-lineage, first identified in the original Ames strain isolated from Jim Hogg County, TX, is descended from the A.Br.001/002 sub-group that has a major presence in most of China. These results suggest a genetic discontinuity between the younger Ames sub-lineage in Texas and the large Western North American sub-lineage spread across central Canada and the Dakotas.

## Background

Ancient Chinese medical books suggest that an anthrax-like disease has been present in China for more than 5,000 years and that by 500–600 A.D. the epidemiology and symptoms of anthrax had been described [1]. A 1995 report from China described the results of an anthrax surveillance and control project in 10 provinces in China between 1990–1994 [2]. Stations in these 10 provinces (Sichuan, Tibet, Inner Mongolia, Xinjiang, Qinghai, Gansu, Guangxi, Guihou, Yunnan and Hunan) reported 72 outbreaks and 8,988 human cases of anthrax. These results, which are indicative of a long history and significant levels of contamination in these specific areas, are the reason for concern by the Chinese Institute of Epidemiology and Microbiology [2].

The population structure of *Bacillus anthracis *has only recently begun to be resolved with specific geographical patterns spread across areas mostly inhabited by man and his animals. Higher genetic resolution within *B. anthracis *has resulted from two molecular typing approaches: An ongoing comparative, single nucleotide polymorphism (SNP) analysis of diverse isolates that describes a conserved, clonally derived basal tree, [3] and a multiple locus variable number tandem repeat analysis (MLVA) system that provides improved resolution among individual isolates [4-7]. This process for molecular typing has now been applied to the study of isolates from China.

An archival collection of 191 *B. anthracis *isolates from China [collection dates from 1947–1983, except isolates A0034 (1993) and A0038 (1997)] was obtained and used in this study (see Methods and Additional file 1). This collection contained an unusual subset of 122 *B. anthracis *isolates recovered from soil, including 107 isolates collected between 1981/1982 in Xinjiang province. This province is located in the western most tip of China and was one of the 10 regions surveyed in the study conducted from 1990–1994. The remaining isolates originated from many regions across the whole of China. This report focuses on the molecular genotyping of these 191 isolates. Our goal was to determine the nature and distribution of genotypes found in China and to establish phylogenetic relationships between these isolates and those found elsewhere in the world.

### Canonical SNP analysis

**Figure 1:**
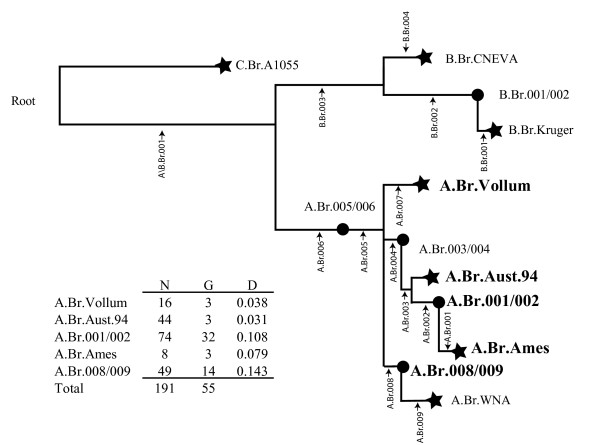
**The twelve canSNP subgroups and sub-lineages of *B. anthracis***. Determined by the analysis of 14 canSNP sites described by Van Ert et al[5]. The five canSNP groups represented in China are indicated in larger and bold fonts in this Neighbor Joining Tree. The number of isolates (N), genotypes (G), and Nei's Diversity Index [8] within groups (D) are illustrated in the table in the lower left. Neighbor-joining trees based upon additional MLVA genotypes within each of these 5 canSNP groups are illustrated in Figures 3 and 5.

The original comparative analysis of 5 *B. anthracis *whole genome sequences examined the status of ~1,000 single nucleotide polymorphisms (SNPs) in 26 diverse isolates [3]. This study revealed an extremely conserved phylogenetic tree with only one homoplastic character in ~26,000 measurements. These results prompted the hypothesis that a few strategically placed "canonical SNPs" could replace the 1,000 assays and still describe an accurate SNP based tree. This idea was confirmed in a study using 13 canonical SNPs (canSNP) to examine 1,000 world-wide isolates of *B. anthracis *[5]. Figure 1 illustrates this original canSNP tree and is used here to define important nomenclature and terminology.

The basic tree is now defined by 7 sequenced genomes that form 7 sub-branches or sub-lineages ending in "stars" in Figure 1. Each of these sub-lineages is designated by the nomenclature from the whole genome sequence site in Genbank, e.g. A.Br Ames, A.Br.WNA (for western North America), and A.Br.Vollum. The relative position of each canSNP is indicated by vertical script and a small arrow and is arbitrarily defined, e.g., as A.Br.001 where A refers to the major subgroup and 001 is the first canSNP (see the A.Br.Ames sub-lineage in Figure 1, also [5]). In this case the derived A.Br.001 SNP defines all isolates that are on the same branch as the sequenced Ames strain. In addition to these 7 sub-lineages the analysis of 26 diverse isolates uncovered 5 nodes or sub-groups along the branches of this tree. Four of these nodes are in the major A Branch and one is in the B Branch (see "circles" in Figure 1). These nodes are defined by the two canSNPs on either side of the node position, e.g. A.Br.001/002 or A.Br.008/009. All of the initial 1,000 isolates in the Van Ert study [5] were placed into one and only one of these 12 sub-lineages or sub-groups.

## Results

### CanSNP analysis of isolates from China

**Figure 2:**
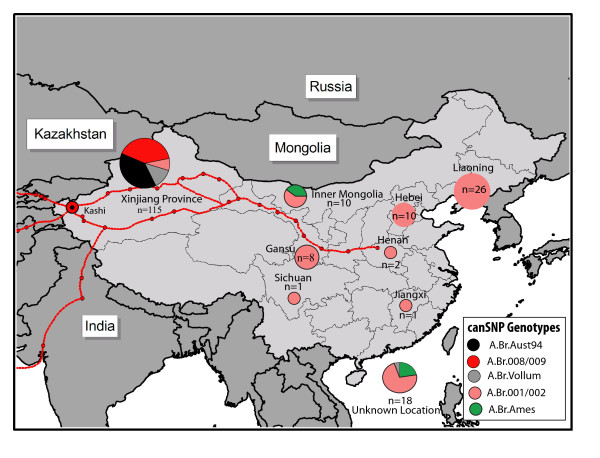
**Geographical distribution of *B. anthracis *isolates in China**. This distribution is based on 12 canSNP genotypes described in Figure 1 and the analysis of 191 isolates from China; also see [5]. The red routes include the western city of Kashi in Xinjiang Province, the main crossroads into China and around the Taklimakan Desert leading into the eastern Chinese provinces.

The 191 *B. anthracis *isolates from China were distributed into only five of these 12 canSNP sub-lineages/sub-groups described by Van Ert et al. [5]. These canSNP groups were A.Br.Vollum, A.Br.Aust.94, A.Br.001/002, A.Br.Ames, and A.Br.008/009 (Figures 1 and 2). Four of the sub-lineages/sub-groups (A.Br.Vollum, A.Br.Aust.94, A.Br.008/009 and A.Br.001/002) were found in the western province of China, Xinjiang (Figure 2). But only isolates from A.Br.001/002 sub-group and the close relative A.Br.Ames sub-lineage were found scattered throughout the other regions of China from east to west. These findings clearly suggest 4 or 5 separate introductions of *B. anthracis *into or out of China, with 3 possibly involving the routes defined as the Silk Road.

The A.Br.008/009 sub-group is a cluster that predominates throughout Europe, the Middle East and China. Xinjiang province had 49 of the worldwide total of 156 A.Br.008/009 isolates (Table insert in Figure 1 and [5]). This province also had 44 of 188 worldwide isolates of the A.Br.Aust94 isolates. This is a sub-group that is also well represented in neighboring Turkey and India. A smaller subset of the A.Br.Vollum sub-lineage (also found in Europe and Africa) accounts for 16 Xinjiang samples out of a worldwide set that totals 48 isolates (Table insert in Figure 1).

The remainder of China is dominated by the A.Br.001/002 subgroup. Chinese isolates represent 74 of the 106 isolates from our worldwide collection of A.Br.001/002 sub-group isolates (Figure 1 and [5]). Only 9 of these isolates are from Xinjiang province to the west. Similarly there are 8 isolates out of 19 worldwide isolates in the A.Br.Ames sub-lineage in the main parts of China.

### MLVA Analysis of A.Br.008/009, A.Br.Aust94 and A.Br.Vollum

**Figure 3:**
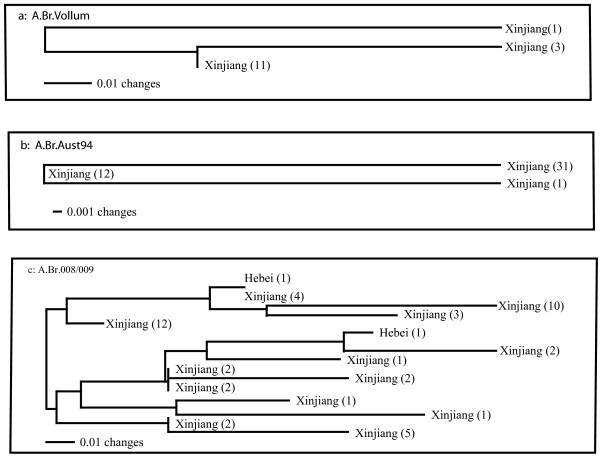
**MLVA15 Analysis of Chinese isolates belonging to the A.Br.Vollum, A.BrAust94 and A.Br.008/009 canSNP sub-lineges/sub-groups**. Representatives of these three sub-groups were only found in isolates recovered in Xinjiang Province, or in unknown locations within China (n = 2). All of these isolates were recovered from soil samples in this province.

CanSNP typing of these isolates has already indicated that there were 49 total Chinese isolates from the A.Br.008/009 subgroup, 44 from the A.Br.Aust94 sub-lineage and 15 from the A.Br.Vollum (Figure 1). Additional sub-typing using 15 MLVA markers indicates that there were only 3 MLVA genotypes within both the A.Br.Vollum (Nei Diversity Index = 0.038 [8]) and A.Br.Aust94 (Nei's Diversity Index = 0.031) sub-lineages but 14 MLVA genotypes within A.Br.008/009 (Nei's Diversity Index = 0.143, Figures 1, 3a, 3b, and 3c). These results suggest repeated infections and outbreaks for each of these sub-groups of *B. anthracis*. The identification of 14 genotypes for the A.Br.008/009 sub-groups is an indication of a combination of possibly repeated introductions and infections and a significantly longer history for this particular clade in this region.

### Branch collapse and ongoing SNP analysis

One of the more remarkable findings from the whole genome SNP analysis of 5 diverse isolates by Pearson et al. [3] was a nearly total lack of homoplastic SNP markers in a query of the status of nearly 1,000 SNP positions in 26 diverse isolates. This finding uncovered a phenomenon called "branch collapse" that resulted in a tree that had no branching except for those created by 7 sequenced reference genomes. The remaining 26 isolates were then either part of one of these seven "sub-lineages" or part of 5 non-branching nodes ("sub-groups") on one of the 7 branches. While the canSNP tree is highly accurate in the typing of 1033 isolates, it lacks resolution because it reflects the results of only 13 of nearly 1,000 SNPs.

**Figure 4:**
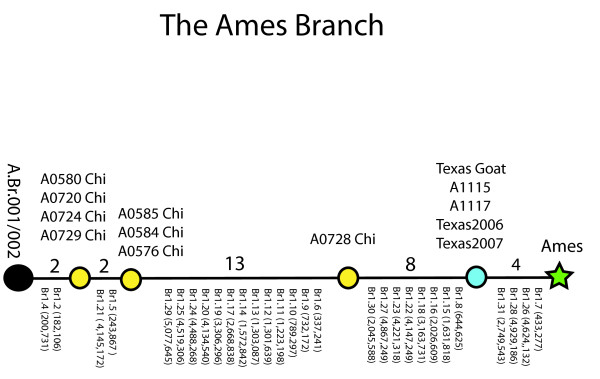
**The Ames branch of *B. anthracis***. This figure shows the relationship between the Ames strain and its closest relatives in a worldwide collection [5]. Twenty-nine of 31 original [5] SNPs are defined by their positions in the Ames genome (NC_003997) and their positions along the Ames branch. Ames has the derived state for all 29 SNPs and the 4 SNPs between Ames and the Texas Goat are specific for the Ames strain alone [5]. A0728 was isolated in China in 1957 but the specific location/source of this isolate is unknown.

Improved resolution between two points was demonstrated by an extensive analysis of the Ames specific branch [9,10] when the status of 29 SNPs that define this branch were determined for the original 12 Ames-like isolates. These analyses have a direct bearing on the isolates from China that are either Ames-like or part of the A.Br.001/002 sub-group (Fig. 1 and 4). The extended analysis of the SNPs on the Ames branch indicate that there are 74 Chinese isolates in the A.Br.001/002 sub-group and 8 additional Chinese isolates (see the table insert in Figure 1) that form three new nodes or collapsed branch points between A.Br.001/002 and the Ames isolate (Figure 4). In addition, there is a fourth node closest to the Ames strain that contains 10 Ames-like isolates from Texas, one goat and 4 bovine isolates [9] shown in Figure 4 and an additional 5 Ames-like isolates from the CDC (Brachman collection, see Methods and Materials). The precise location for the recovery of these latter isolates is unknown except that they originated in Texas. These 19 isolates (8 Chinese, 10 Texas) and the Ames strain represent a highly resolved, SNP based A.Br.Ames sub-lineage. These results indicate that the original Ames strain and a subset of 10 Texas isolates are decendents of a rare lineage that is otherwise only found in China.

### MLVA: A.Br.001/002

**Figure 5:**
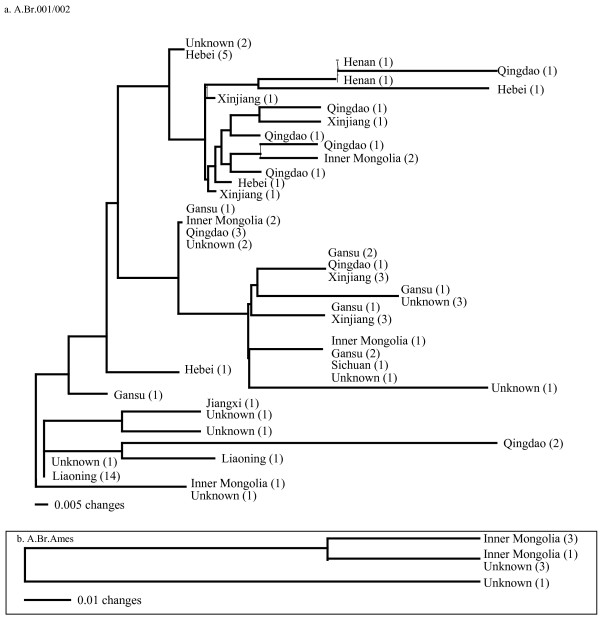
**MLVA 15 Analysis of A.Br.001/002 and A.Br.Ames sub-group and sub-lineage respectively**. The A.Br.001/002 sub-group has a relatively large diversity index (See Figure 2) and suggests that this sub-group has a long history in China with repeated outbreaks and eventual spread throughout much of the country.

The 15 marker MLVA analysis (MLVA15) of the 74 isolates belonging to the A.Br.001/002 sub-group yielded 32 different genotypes (Nei Diversity Index = 0.108, Figures 1, 5a). This high diversity index is an indication that this sub-group, spread throughout the whole of China (Figure 2), is another sub-group of *B. anthracis *with a long and extensive evolutionary presence in China.

## Discussion

Human anthrax has been an old and continuous problem in many rural regions in China where as much as six percent of environmental samples have been found to be contaminated with *B. anthracis *[2,2]. An archival collection of 191 *B. anthracis *isolates was obtained from China and canonical SNP typing indicated that only 5 of the 12 worldwide sub-lineages/sub-groups of this pathogen were represented in this collection. One striking feature of the distribution of these *B. anthracis *isolates within this country was the discovery that three of the five canSNPs sub-lineages/groups (A.Br008/009, A.BrAust.94, and A.Br.Vollum) are predominantly found in the western most Chinese province of Xinjiang. The previous observation [5] that these three sub-lineages/sub-groups are prominent genotypes in India, Pakistan, Turkey and most of Europe suggest a likely transmission pattern for anthrax along the ancient trade route known as the Silk Road [11] that extended from Europe, the Middle East, portions of Asia and into Xinjiang province and the whole of China, Figure 2.

More specifically, 107 isolates were recovered from "soil samples" between 1981–1982 from unspecified sites relatively close to the city of Kashi in this province. Kashi (also Kashgar, Kaxgar, Kǝxkǝr) was a major "oasis" crossroads city along the ancient Silk Road and dates back more than 2,000 years [11]. Consistent with the idea that the life cycle of *B. anthracis *can be maintained by viable spores in previously contaminated areas, the later 1990–1994 surveillance project in China described three regions in Xinjiang Province where severe anthrax outbreaks had previously occurred [2]. Two of these towns, Zepu and Atushi, are located approximately 144 and 33 kilometers respectively from the city of Kashi. In the 1990–1994 study, Zepu recorded 24 villages with 202 human infections and Atushi recorded 4 villages with 81 human infections.

Despite a clear correlation between canSNP genotypes from the A radiation and the spectrum of isolates found across the Trans-Eurasian continents, there is one set of genotypes in Europe that are clearly missing in China. These are representatives from the B branch that appear to be prevalent in several European states including at least 27 B2 isolates from France and isolates identified in both the B2 and B1 branches from Croatia, Germany, Poland, Italy, Norway and Slovakia [5,6,12]. It is not obvious why examples of the B branch are limited mostly to Africa, this region of Europe and a small location in California, USA. Aside from sampling issues the B branch does not appear to have participated in the world-wide, dynamic radiation that has characterized the A branch [5].

Additional analyses with the rapidly evolving MLVA markers suggest that establishment in China of two of these sub-groups/sub-lineages, A.Br.Aust94 and A.Br.Vollum, resulted from relatively recent events (Figure 3a and 3b). In both of these instances, a sizeable number of isolates (44 and 15, respectively) are clustered into only three different MLVA15 genotypes (Nei's Diversity Indices = 0.031 and 0.038 respectively, Figure 2). Although these results may reflect a certain sampling bias, the MLVA comparison to other worldwide isolates from this branch indicates that the A.Br.Aust94 sub-lineage in China is most closely related to isolates recovered from the large 1997 outbreak in Victoria, Australia (data not shown). The precise origin and time-scale for this exchange is not certain but relatively recent exchanges between the Far East and Australia appear to have originated from India [13], which could represent a common ancestor or an intermediate step in the transmission route.

By direct contrast the MLVA analysis of 49 isolates belonging to the A.Br.008/009 sub-group revealed a more complex pattern with 14 different MLVA15 genotypes (Nei Diversity Index = 0.143, Figures 1 and 3c). This is a remarkable finding because it indicates that a variety of MLVA genotypes are persisting in the different soils from which the A.Br.008/009 isolates were recovered. These results are an indication that A.Br.008/009, a major sub-group in Europe and Asia [5], has had an extensive history in China. It is difficult to determine the precise origins of the A.Br.008/009 subgroup (e.g. China versus Europe) at this point because rapidly evolving MLVA markers are subject to homoplasy and potentially inaccurate phylogenetic reconstructions. These issues can eventually be resolved using additional whole genome sequencing and phylogenetic inference to more accurately predict the origins of the A.Br.008/009 sub-group.

The Ames sub-lineage appears to have descended from the A.Br.001/002 sub-group, a sub-group that has 106 isolates in our worldwide collection [5]. Seventy-four of these accessions were isolated from outbreaks in China and the remaining 32 isolates were recovered in the UK, other parts of Europe, North America and other parts of Asia. The large number of MLVA15 genotypes (n = 32) among the 74 Chinese isolates and a wide distribution throughout the country indicates that the A.Br.001/002 sub-group is a major part of the *B. anthracis *population structure in this region (Figure 5a). This sub-group also appears to be basal to the Ames sub-lineage, indicating that 8 isolates from China and 11 isolates from Texas may share common ancestors that originated in China (Figure 5b and [10]).

**Figure 6:**
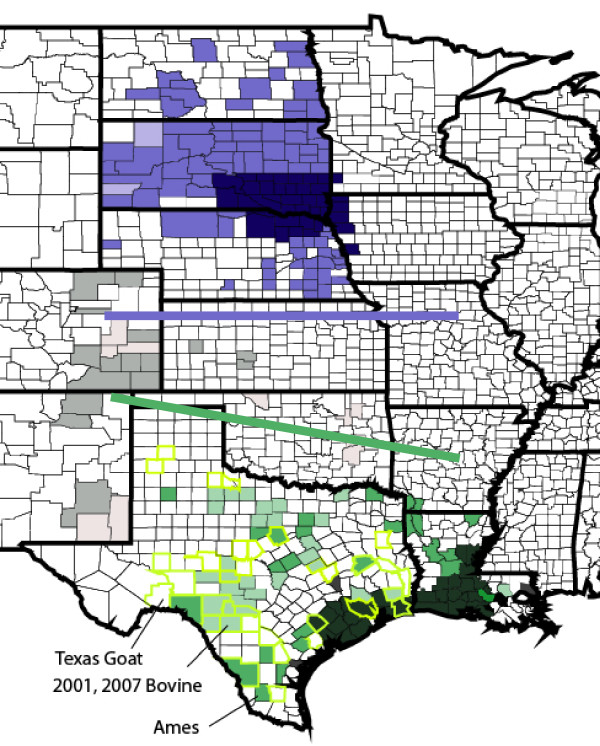
**Historical Anthrax Incidences between 1915–1944 in Texas/Louisiana and The Dakotas/Nebraska/Iowa**. Adapted from Stein (1945, [15]). Darker colors represent severe outbreaks and the lighter colors represent sporadic outbreaks. The blue and green colors were used to illustrate that two distinct genotypes (Western North America (WNA) and the Ames sub-lineage) have been indentified in "modern" isolates from these two regions. The counties bordered in yellow in Texas indicate counties where documented incidents of anthrax have occurred between 1974 and 2000. The numbers 1–4 indicate the counties in which the original Ames strain, 2 bovine samples and a goat sample have been analyzed by current genotyping methods as belonging to the Ames sub-lineage. The molecular analysis of more than 200 isolates from North and South Dakota indicates a pre-dominance of the sub-lineage WNA in this region. The gray colors indicate moderate to sparse outbreaks in the states adjoining the Dakotas and Texas.

How then did the Ames lineage come to Texas and why is this lineage not found in Europe? This is still not known and subject to considerable speculation. By several accounts, it is believed that anthrax was introduced into the Gulf Coast states (Louisiana and Texas) by early settlers from Europe. Stein [14,15] indicates that the first recorded episodes of anthrax in livestock in Louisiana occurred in 1835, 1851 and 1884; and in Texas in 1860 and 1880. By 1916, when a first national survey was conducted to obtain nation-wide information on the incidence of anthrax, Texas already had 41 counties reporting infections. A composite of outbreaks compiled after the 4^th ^National Survey by the U.S. Department of Agriculture between 1916–1944 (Figure 6) indicates three major outbreak pockets: one in California, one in the Dakotas/Nebraska and the third along the coastal regions of Texas and Louisiana [15].

An important feature of the outbreaks in Texas is that the "modern" outbreaks have occurred repeatedly in many of the same counties depicted in this historical map (Figure 6 and USDA Report: Epizootiology and Ecology of Anthrax: http://www.aphis.usda.gov/vs/ceah/cei/taf/emerginganimalhealthissues_files/anthrax.pdf). A culture-confirmed study between 1974–2000 indicated that 179 isolates were spread across 39 Texas counties (counties outlined in yellow) that are in general agreement with the dispersal patterns observed in the early national surveys depicted in Figure 6. The one significant difference is a shift from the historical outbreaks in the coastal regions to counties more central and southwesterly in "modern" times. Similarly, culture-confirmed isolates from a 2001 outbreak in Val Verde, Edwards, Real, Kinney and Uvalde counties in southwest Texas are similar to outbreaks in 2006 and 2007 when 4 Ames-like isolates were recovered from Real, Kinney, and Uvalde county [9].

It appears that *B. anthracis *was introduced into the Gulf Coast, probably by early European settlers or traders through New Orleans and/or Galveston during the early to mid 1800s. The disease became established along the coastal regions and then became endemic to the regions of Texas where cattle and other susceptible animals are currently farmed. Are these *B. anthracis*, Ames-like genotypes from the Big Bend region (Real, Kinney, Uvalde counties) of Texas representative of the ancestral isolates brought to the Gulf Coast? Van Ert et al. [5] used synonymous SNP surveys to estimate the divergence times between the major groups of *B. anthracis *and these estimates suggest that the Western North American and the Ames lineages shared common ancestors between 2,825 and 5,651 years ago. Extrapolating to the much shorter SNP distances between the most recent Chinese isolate (A0728) and the recent Texas isolates on the Ames sub-lineage would approximate that these two shared a common ancestor between 145 to 290 years ago. These estimates would be consistent with the hypothesis that an Ames-like isolate was introduced into the Galveston and/or New Orleans area in the early to middle 1800s.

This relatively recent expansion is in direct contrast to analyses of the Western North American (WNA) sub-lineage that appears to have an ancient and significantly longer evolutionary presence in North America; this group stretches from the central regions of Canada and into North and South Dakota (Figure 6; [16]). Phylogenetic reconstruction of > 250 Western North American isolates indicates that the more ancestral isolates of this sub-lineage are found in the upper reaches of central Canada and portrays a migration pattern where the youngest isolates are found in cattle outbreaks in North/South Dakota and Nebraska. Kenefic, Pearson et al. [16] suggest that the ancestral isolates may have entered the North American continent via the Beringian straights 13,000 years ago.

A recent ecological niche model suggests that natural anthrax outbreaks are "concentrated in a narrow corridor from southwest Texas northward into the Dakotas and Minnesota" [17]. This model indicates that conditions like vegetation, precipitation and altitude along this corridor are suited for maintaining naturally occurring anthrax outbreaks in livestock and wildlife. Although historical records provide evidence that validate this model, there is a molecular and genotyping anomaly: there does not appear to be a direct epidemiological link between the "younger" Ames-like cluster and the Western North American lineage. Despite nearly 100 years of monitoring since the first national outbreak tabulations [15], there is still a clear physical division between the Ames-like isolates to the south and the Western North American lineage to the north (Figure 6). This gap is not obvious until the spatial patterns are examined in hindsight of the genetic discontinuity. These observations probably reflect the awareness and controls that were being observed for anthrax outbreaks as the US entered the 20^th ^century.

Limited sample analysis of isolates from the Texas/Louisiana coastline prevents any conclusions about the overall dominance of the Ames sub-lineage in this area and we also cannot exclude the possibility that there are other sub-groups/sub-lineages that might have been imported and even become transiently established along the Texas/Louisiana Gulf region during this same time frame.

## Conclusion

Despite containing only 5 of the initial 12 canSNP genotypes used to define a collection of world-wide isolates [5], the analysis of 191 Chinese *B. anthracis *isolates reveals an interesting impact on global distribution. The major diversity in these isolates is concentrated in the western province of Xinjiang and especially the city of Kashi, the hub of the Silk Road around the Taklimakan Desert into and out of China. These results reinforce the idea that this Silk Road region was central to the spread of anthrax between the trans-Eurasian continents.

In addition to the three distinct sub-groups found in the western Xinjiang province, the central and eastern regions of China are dominated by a different, highly diverse, canSNP sub-group, A.Br.001/002. This sub-group is a major presence in relationship to our world-wide collection since 70% of all the isolates and most of the diversity for this sub-group were in this Chinese collection. These results suggest that the A.Br.001/002 cluster may have originated in China. Finally, the Ames and Ames-like strains in Texas are descended from common ancestors in Inner Mongolia in China as an extension of this sub-group. It is curious that this lineage would become established in Texas, and perhaps Louisiana, and not in Europe. This leaves behind a missing historical gap within the phylogeography of the Ames lineage.

## Methods

### *B. anthracis *isolates

The 191 *B. anthracis *isolates from China used in this study were previously isolated from a variety of sources and provinces in China (see Additional file 1). One hundred and fifteen isolates were from Xinjiang Province in western China including 107 isolates from soil samples. The remainder of the isolates were recovered from the following provinces with the number of isolates in parenthesis: Hebei (10), Gansu (8), Henan (2), Inner Mongolia (10), Jiangxi (1), Liaoning (26), Sichuan (1) and 18 isolates where the province of origin was not known. In addition to the 107 soil samples from Xinjiang Province isolates were obtained from the following sources: soil (15 additional), air (4), bovine (3), buffalo (1) fur (2), human (25), laboratory (1), marmot (1), sheep (3), swine (3) and unknown sources (26). In addition to the Chinese isolates there are 6 isolates that were used to describe Figure 4[9,10] and an additional 5 isolates that were obtained from the CDC as part of the "Brachman Collection" (CDC ID # 34064, 34279, 402, 482, 490). All 11 of these isolates belong to the Ames sub-lineage and all were isolated in Texas between 1959–2007. This analysis also includes the original Ames strain that was isolated in 1981 from bovine in Jim Hogg County.

All isolates were initially genotyped for a *B. anthracis *species-specific *plcR *nonsense mutation that has been suggested as being necessary for stabilization of the virulence plasmids [18]. This single nucleotide polymorphism appears to be diagnostic for *B. anthracis *[19]. In this study the ancestral state for this marker was used to root the *B. anthracis *SNP tree to the older and more diverse *B. cereus/B. thuringiensis *tree. DNA was isolated from each of the 191 isolates as previously described [5].

### CanSNP Genotyping

TaqMan™ -Minor Groove Binding (MGB) allelic discrimination assays were designed for each of 13 canSNPs and have been described in great detail by Van Ert et al. [5]. The genomic positions for each canSNP and the primer sequences and probes for each site can be found in Supplemental Tables 4 and 5 in the Van Ert et al. [5].

### MLVA Genotyping

Multiple Locus Variable Number Tandem Repeat (VNTR) Analysis (MLVA) was used to determine the overall diversity of the isolates within each sub-group and sub-lineage. The first 8 marker set used in this analysis were initially described by Keim et al., [4] and a second set of 7 additional markers were described by Zinser [20]. This 15 marker, high-resolution, MLVA system is described in detail by Van Ert et al. [5] with the genomic positions and primer sets for these assays described in Supplemental Tables 2 and 6 of this reference.

### Phylogenetic Inference

The genetic relationships among the Chinese isolates were established using a hierarchical approach where the slowly evolving, highly conserved, canSNP markers were first used to place each isolate into its appropriate clonal lineage. The 15 more rapidly evolving, VNTR loci, were then used to measure the genetic diversity and to determine the number of specific genotypes within each of these clonal lineages. Neighbor joining phylogenetic trees were constructed for both the canSNP and MLVA datasets using PAUP (Phylogenetic Analysis Using Parsimony) [21]; and the MEGA 3 software package [22] was used to calculate average within group distances for each of the five canSNP sub-groups/sub-lineages.

## Authors' contributions

TSS: Conception, acquisition and analysis of data, interpretation of data, drafting of manuscript, approved final draft. RTO: Analysis and interpretation of data, drafting of manuscript, approved final draft, BW: Acquisition and interpretation of isolate data, approved final draft, RE: Acquisition and interpretation of DNA signature data, approved final draft, LYH: Acquisition and interpretation of DNA signature data, approved final draft, JMUR: Acquisition and interpretation of DNA signaturedata, approved final draft, MD: Acquisition and interpretation of DNA signature data, approved final draft, SRZ: Acquisition and interpretation of DNA signature data, approved final draft, LJK: Provide insight for relationship between worldwide and Chinese isolates, approved final draft, JB: Acquisition and interpretation of data, approved final draft, JMS: Acquisition and interpretation of data, approved final draft, TP: Input on phylogenetic analysis of datasets, draft manuscript, approved final draft, DMW: Provide insight into geographical relationships between worldwide isolates, draft manuscript, approved final draft, AH: Provide data and genotyping information for new Texas isolates belonging to Ames sub-lineage, approved final draft, JR: Initial sequencing, assembly and analysis of genomes, approved final draft.

PK: Responsible for concepts, vision and direction for the entire project, draft manuscript, approved final draft.

## Supplementary Material

Additional file 1 **List and description of isolates including the canSNP and MLVA Genotypes for each isolate**. This table contains: The Keim Laboratory ID # for each isolate, the year of isolation, the source, the canSNP ID, and the originating province. This information is followed by the Keim Genetics Laboratory 15 MLVA genotypes for each isolate, see supplemental material from Van Ert et al., [5]. Click here for file
