# Supplementary figures and images for: Bacillus anthracis in China and its relationship to worldwide lineages
Source: BMC Microbiol. 2009 Apr 15;9:71. doi: 10.1186/1471-2180-9-71 (PMC2674057; doi:10.1186/1471-2180-9-71)

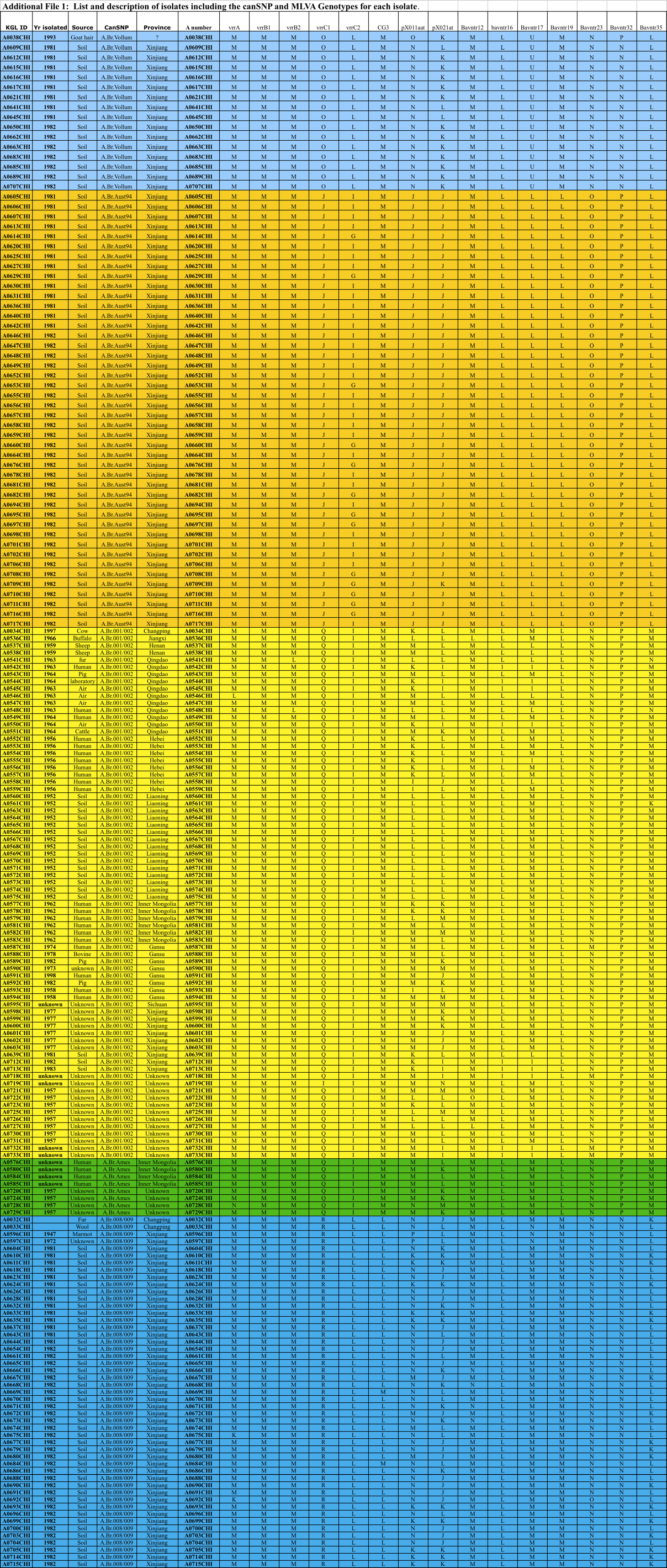

Supplement: Additional file 1 — List and description of isolates including the canSNP and MLVA Genotypes for each isolate. This table contains: The Keim Laboratory ID # for each isolate, the year of isolation, the source, the canSNP ID, and the originating province. This information is followed by the Keim Genetics Laboratory 15 MLVA genotypes for each isolate, see supplemental material from Van Ert et al., [5]. [file 1471-2180-9-71-S1.doc]
